# The Effects of Quality Assurance System Implementation on Work Well-Being and Patient Safety: Protocol for a Mixed Methods Study

**DOI:** 10.2196/45200

**Published:** 2023-11-23

**Authors:** Anni Vuohijoki, Mira Huusko, Leena Ristolainen, Pipsa Hakasaari, Hannu Kautiainen, Juhana Leppilahti, Sanna-Maria Kivivuori, Heikki Hurri

**Affiliations:** 1 Faculty of Medicine University of Oulu Oulu Finland; 2 Research Institute Orton Helsinki Finland; 3 Finnish Education Evaluation Centre Helsinki Finland; 4 Primary Health Care Unit Kuopio University Hospital Kuopio Finland; 5 Folkhälsan Research Center Helsinki Finland; 6 Translational Medicine Research Unit Medical Research Center Oulu Oulu University Hospital Oulu Finland; 7 Helsinki University Hospital Helsinki Finland

**Keywords:** Joint Commission International, health and safety, JCI, human resource management, organizational development, quality in health care

## Abstract

**Background:**

Systematic monitoring of work atmosphere and patient safety incidents is a necessary part of a quality assurance system, particularly an accredited system like the Joint Commission International (JCI). How the implementation of quality assurance systems affects well-being at work and patient safety is unclear. Evidence shows that accreditation improves workplace atmosphere and well-being. Thus, the assumption that an increase in employees’ well-being at work improves patient safety is reasonable.

**Objective:**

This study aims to describe the protocol for monitoring the effects of implementing the quality assurance system of JCI at Orton Orthopedic Hospital on employees’ well-being (primary outcome) and patient safety (secondary outcome).

**Methods:**

Quantitative (questionnaires and register data) and qualitative (semistructured interviews) methods will be used. In addition, quantitative data will be collected from register data. Both quantitative and register data will be analyzed. Register data analysis will be performed using generalized linear models with an appropriate distribution and link function. The study timeline covers the time before, during, and after the start of the accreditation process. The collected data will be used to compare job satisfaction, as a part of the well-being questionnaire, and the development of patient safety during the accreditation process.

**Results:**

The results of the quality assurance system implementation illuminate its possible effects on the patient’s safety and job satisfaction. The repeatability and internal consistency reliability of the well-being questionnaire will be reported. Data collection will begin in May, 2024. It will be followed by data analysis and the results are expected to be published by 2025.

**Conclusions:**

The planned study will contribute to the evaluation of the effects of JCI accreditation in terms of well-being at work and patient safety.

**International Registered Report Identifier (IRRID):**

PRR1-10.2196/45200

## Introduction

### Overview

Systematic monitoring of work atmosphere and patient safety incidents is a necessary part of a hospital’s quality assurance systems, particularly an accredited system like the Joint Commission International (JCI) [1,2]. Accreditation and related education have been shown to increase motivation and improve work atmosphere and awareness of patient safety. Statistically significant improvements have been noted in defect management (particularly in the context of instrument cleaning and disinfection effectiveness) in a randomized controlled trial evaluating the impact of JCI accreditation [3]. Physician-nurse relations as well as management-line staff relations have been reported to improve along accreditation [4].

Accreditation is a costly and time-consuming process [5], causing a legitimacy problem for a hospital’s management [6]. This situation raises a fundamental question: Is accreditation worth pursuing considering the time and effort it requires? Thus, further research is needed from a different view. How the implementation of quality assurance systems affects well-being at work and patient safety is not studied extensively [7]. The effects of quality assurance systems on work atmosphere and patient safety are challenging to determine due to numerous potentially confounding factors [8,9]. However, assuming that well-being at work and work satisfaction are strongly correlated with good outcomes in health care service delivery, including patient safety, is reasonable. This assumption is supported by Schwarz et al [10], who found that work-life integration among health care workers is associated with improvements in teamwork and safety across various safety culture domains [10]. In a study by Lopes et al [11], job satisfaction and safety culture were directly proportional. Better working conditions can provide a virtuous cycle of patient safety and occupational health [11].

**Figure 1:**
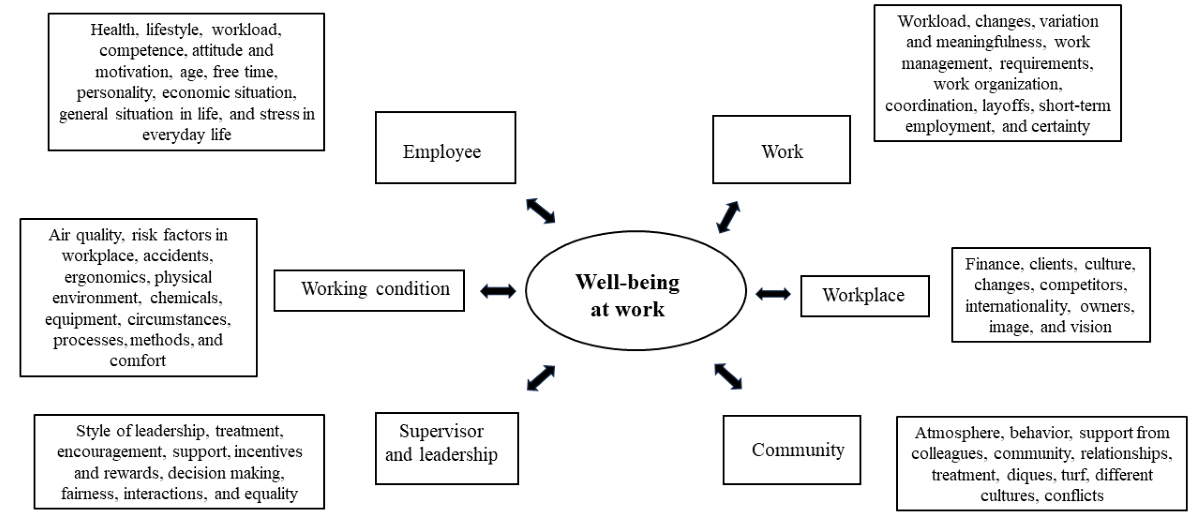
Domains well-being at work by Naumanen (2018) [14].

The Finnish Institute of Occupational Health defines well-being at work as safe, healthy, and productive work, which is carried out by competent employees and work communities in a well-managed organization. Employees and work communities experience their work as meaningful and rewarding, and they consider work to support their life management [12]. The importance of well-being at work is highlighted in health care services, as labor input is directed toward patients. Well-being improves work performance, further promoting good workplace outcomes. For instance, well-being at work reduces the risks of exhaustion, workplace accidents, illnesses, and the loss of tacit knowledge regarding drug abuse [13]. The well-being of the working community is comprised of the performances of its members and linked to factors beyond each employee’s performance and influence. In this study, the operationalization of well-being is based on a model that considers worker health, subjective well-being, capabilities, motivation, the organizational management model, management workload, company values, and the overall atmosphere as well as the conditions of the work environment, such as work content and instruments with which the work is executed (Figure 1) [14].

### Aim and Objectives

This protocol will describe the procedure and methods for monitoring the effects of implementing the quality assurance system of JCI at Orton Orthopedic Hospital on employees’ well-being (primary outcome) and patient safety (secondary outcome). The system implementation effects will be evaluated through work-related well-being surveys using direct and indirect evaluations, and patient safety will be evaluated using register data.

## Methods

### Overview

The study sample will include about 200 employees working in a hospital. The hospital is a private orthopedic hospital focused on joint replacements and back surgery and has a rehabilitation center. This hospital is owned by a large public health care hospital, which also performs JCI implementation. Table 1 illustrates the accreditation process and its monitoring. Textbox 1 [15] presents the registry data collection system. The implementation will begin following a JCI orientation training organized by JCI. The company must organize their employees during the process. The JCI procedure is designed to be ready in 4 years. Baseline surveys (questionnaires and interviews) will be conducted, and all surveys will be repeated when the implementation is 50% complete.

**Table 1:** Methods and data collection system during the accreditation process.

Baseline surveys^a^	Data collection system
Questionnaires	A total of 35 questions; registering and monitoring the well-being of the employees of the Orton Orthopedic Hospital Repeatability test of the questionnaires
Register data	HaiPro^b^ Absence due to illness Employee turnover Injuries of the employees Cost of occupational health case Patient insurance cases Rate of wound infections Revisions Time of surgery
Qualitative method (semistructured interviews)	A total of 4 groups (doctors, nurses, therapists and administration, and support staff)

^a^Surveys, including questionnaires and register data, as well as qualitative methods (semistructured interviews) will be repeated after half of the implementation and after finishing accreditation.

^b^HaiPro is a web-based tool for reporting patient safety incidents. Anonymous and voluntary input ensures confidential reporting and processing of the safety incidents. The HaiPro method does not seek to blame [15].

**Register data collection 2 years prior to implementation, when the implementation process is 50% complete, and when the facility is ready for accreditation. A web-based tool (HaiPro) will be used for reporting patient safety incidents and risk events, which are divided into near-miss and injurious events.**

**Register data collection**

- HaiPro—near-misses and misses (n)
- Absence due to illness (days)
- Employee turnover (n)
- Employee injuries (n)
- Cost of occupational health care (€/year)
- Patient insurance cases (n)
- Rate of wound infections (n)
- Time per surgery (h/min)
- Revision surgery (%)

### Quantitative Studies

#### Questionnaires

This study applies a mixed methods approach, including quantitative (questionnaires and register data) and qualitative (semistructured interviews) methods [16]. The study uses a convergent design, where both qualitative and quantitative data are collected as well as analyzed and results are reported at the same time [17]. The occupational well-being questionnaire in this hospital was developed before the planned study to facilitate good personnel management. In the questionnaire, job requirements and job resources have formed the theoretical framework of the survey [18-20]. It also included 4 questions designed to capture the participants’ views on leadership, values, expertise, and work atmosphere, based on Otala and Ahonen [21].

The questions are divided into 3 sections: questions 1-6 ask for the participants’ background information, questions 7-31 focus on physical and psychological well-being and connect to functional capacity, and questions 32-35 concern work satisfaction (Multimedia Appendix 1). The first section ascertains the employees’ health and well-being regarding work burden and the effects of illnesses and strains on functional capability. The questions will be evaluated on a 10-point Numeric Rating Scale (1=no work-related problems; 10=completely unable to work). Questions 7-21 evaluate perceived health and mood, distress, anxiety, and the ability to take care of oneself on a 3-point Likert scale (1=no problems; 3=completely unable). These questions are evaluated on a 5-point Likert scale (1=completely agree; 5=completely disagree).

In the third section, question 32 describes management from the viewpoint of an immediate superior. Question 33 explores the know-how viewpoint, aiming at charting the perspective to improve one’s competence and knowledge. Question 34 focuses on company values and acting upon them. Question 35 concerns team spirit, which is one of the company’s most important values. Questions 32-35 are evaluated on a 5-point Likert scale (1=completely agree; 5=completely disagree).

#### Register Data

Register data, including the employees’ sick leave and work accidents, will be collected from 2 years prior to implementation to 2 years after implementation. We will also use a web-based tool (HaiPro) for reporting patient safety incidents, which are all treatment events that either harmed or could have harmed them [15]. HaiPro is a web-based tool for reporting patient safety incidents. Anonymous and voluntary input ensures confidential reporting and processing of safety incidents and risk events. These events are called risk events and are divided into near-miss and injurious events [15]. In addition, the number of reoperations, the rate of wound infections, surgery times, and notices of injuries reported to the Finnish Patient Insurance Center will be collected. Textbox 1 shows the register data collection during the accreditation process.

### Statistical Analysis

Primary and secondary end point analysis will be performed on all participants receiving 1 or more administrations of study treatment (the intent-to-treat population). However, no sample size calculation will be performed in advance due to the unavailability of accurate and reliable data for making such estimates. We will include all individuals who will respond to the questionnaire in this study. The descriptive statistics are presented as means (SDs), medians (IQRs), or counts with percentages. The groups will be compared using the *t* test, permutation test, and ANOVA for continuous variables and the Pearson chi‐square test or Fisher exact test for categorical variables. Register data analysis will be performed using mixed-effect linear models with an appropriate distribution and link function; models do not require complete data and can be fit even when individuals or data do not have observations at all time points (missing data will be handled by using available-data analysis) [22]. Confounding factors will be adjusted. Models include age, sex, working time, education years, and employment status. In case there are violations of the assumptions or theoretical distribution of the test statistics (eg, nonnormality, skewness, kurtosis, or a small number of observations), a bootstrap- or permutation-type analysis will be used.

Traditional test theory analyses will be used to verify the reliability and validity of the questionnaire. These analyses include internal consistency using Cronbach α, inter-item correlations, and exploratory factor analysis (using the iterated principal-factor method for factoring and oblique promax-rotated factor loadings on polychoric correlation matrix). The test-retest reproducibility will be evaluated by intraclass correlation coefficients using the 1-way random effects model. Coefficients of reproducibility and their 95% CIs will be also calculated. Correlation coefficients will be calculated by the Pearson or Spearman method. Normal distributions will be evaluated graphically and with the Shapiro-Wilk W-test. Stata 17.0 (StataCorp LP) will be used for the analysis.

### Qualitative Studies

Participants in the focus group interviews include working community members. The groups consist of multiprofessional individuals, and the interviews last about an hour. These qualitative interviews aim to broaden the baseline views, conceptions, and expectations of the personnel regarding quality assurance and their well-being before implementing the JCI quality assurance system. The semistructured interviews will be conducted by an external interviewer with knowledge of different quality assurance systems and conducting qualitative research.

Qualitative data are collected in 3 thematic group interviews that feature 12 participants. To create the groups for the interviews, a list of the hospital’s employees will be collected and grouped into 4 categories: physicians; nurses; therapists, other medical staff, and the administration; as well as support staff and secretaries. The personnel will be listed in each category in alphabetical order. Consequently, random selections will be made from the staff at the hospital, stratified by occupation, for all groups. After the draw, each employee can opt out of their assigned group. In such cases, the group is supplemented with the next person on the list. The researchers conducting this study are exempted from the lists. After 3 semistructured focus group interviews, the main themes will presumably recur, and the point of saturation will be reached [23].

The interviews will be recorded with the participant’s permission, and the tapes will be transcribed subsequently. The interviews will be analyzed using qualitative content analysis, a conventional content analysis [24]. The analysis aims to gain an understanding of how individuals in the small groups perceive the quality assurance system, assess its usefulness, and recognize the need for its development, all from the perspectives of different employees. The semistructured interviews ask participants about their expectations of the quality system, their involvement in the JCI orientation, as well as the perceived benefits and drawbacks of the quality system. In addition, the focus group interviews will ask about staff experiences in relation to safety, well-being, and motivation at work as well as the prospects of the hospital. The interviews will conclude with questions on quality factors related to surgery (eg, surgery times), patient safety, and experiences of patient care. Textbox 2 includes the semistructured interview questions.

The content of the open-ended questions will be coded according to the themes emerging from the data. The most prevalent themes will be selected and reported in the study. These themes will help explain and deepen the understanding of the responses to the quantitative approach of the study in describing the perceived conceptions of the quality system and its relationship to occupational safety, patient safety, and surgery.

The interviews will be renewed after half of the implementation is completed. The same groups will be requested to participate in the interviews; however, certain community members may change their working place or may not participate in the renewed test. Those members will be replaced by other members of the working community. If the point of saturation is not reached in the midterm interviews after 3 interviews, 4 or more interviews will be conducted [23].

**Semistructured interview questions.**

**Expectations from the quality assurance system, participation in orientation, perceived benefits of the quality assurance system, results, and their quality**

- How have you participated in the quality assurance work?
- How have you experienced the training related to the quality assurance system and other orientations?
- What expectations do you have for the quality assurance system (ie, Joint Commission International)?
- What kind of benefits can the quality assurance system have? Do you think it might have some disadvantages as well?
- What kind of results can the quality assurance system have?
- How does it affect the quality of activities in the hospital?
- What kind of changes does the quality assurance system bring to your everyday work?
- In what state do you think the quality assurance system is right now?

**Occupational safety of personnel, work motivation, and prospects of the hospital**

- How does the quality system affect occupational safety?
- What about work motivation?

**Occupational well-being**

- What do you perceive as factors related to well-being at work?
- What are the driving forces of occupational well-being in our hospital?
- What kind of effects the quality assurance system can have on well-being at work?
- Which aspects of occupational well-being are affected by the quality assurance system?

**Factors related to surgery (eg, surgery times), patient safety, and patient care**

- What kind of effects does the quality assurance system have on factors related to surgeries?
- What kind of effects does the quality assurance system have on surgery times?
- What about patient safety?
- What other effects does the quality system have in relation to patient care?
- How do you think the quality assurance system will affect the future of the hospital?
- What else would you like to say about the quality assurance system or occupational well-being?

### Ethical Considerations

The study was submitted to the University of Oulu Human Sciences Ethics Committee. An ethical review was not required based on their criteria. Participants will provide their written consent for the interviews.

## Results

The results of the quality assurance implementation will illuminate the possible effects on patient safety and job satisfaction. The repeatability and internal consistency of the well-being questionnaire will be reported. The qualitative research results describe the employees’ experiences with the quality implementation process. Data collection will begin in May, 2024. It will be followed by data analysis and the results are expected to be published by 2025.

## Discussion

### Expected Outcomes

The Finnish Institute of Occupational Health defines well-being at work in 3 different ways: the first one is based on doing in the sense of accomplishing, the second emphasizes experience, and the third provides a description of the experience [25]. Well-being results from the fulfilment of the important needs of individuals and the realization of goals and plans set for one’s life. Goal-oriented activity and commitment to tasks lead to well-being. However, the history of well-being at work is short. The concept of quality and productivity in working life has only recently evolved and includes aspects such as learning and social activities [25]. Well-being at work is also subject to various perspectives in different countries and cultures and even in different workplaces within the same country. That is why we believe it is crucial that in the planned study, we use a customized questionnaire for well-being at work; this questionnaire will encompass generic items from the Finnish working life context and will be supplemented with specific questions that are important for Orton Orthopedic Hospital.

The need for quality assurance systems in health care is widely accepted. However, the added value of an accredited quality assurance system has not been widely studied. When undertaking such a large task, the effects of pursuing an accredited quality assurance system should be evaluated. The analysis will be challenging in a comparative study design due to practical and ethical reasons, although a randomized controlled trial published recently offers some insight [3]. Therefore, in this study protocol, a quasi-experimental study design will be followed [26], with a focus on employee well-being and patient safety, under the assumption that they are interlinked. Therefore, procedures and practices will be clarified for workers to diminish errors and increase patient safety. The added value of branding and marketing will be ignored, but accredited quality influences these issues as well.

In this protocol, the primary and secondary outcomes are well-being at work and patient safety, respectively. Patient safety has 4 main items: all the HaiPro notices [15], the number of wound infections, the number of patient insurance cases, and the number of revision surgeries. Certain overlaps exist between these items and must be considered in the analysis. Worker well-being register data will also be collected, including information on illness-related absences, employee turnover, employee injuries, and the costs of occupational health care.

A staff survey is an efficient way to learn the views of all personnel. Surveys also help employees evaluate their efforts and input [27]. Our study will use various evaluations. The most important variable is employee well-being, which is evaluated through the questionnaire. This questionnaire is specifically developed for the study and complemented with the register data follow-up regarding sick leave, employee turnover, and costs of occupational health care.

We acknowledge the problem of presenteeism, which involves the act or culture of employees continuing to work as a performative measure, despite negative consequences); this is a rising issue in the workplace. If left unrecognized and unresolved, presenteeism can lead to poor well-being, low morale, burnout, and increased employee turnover. It also poses a huge threat to productivity because employees who show up to work when they are not feeling mentally or physically well cannot provide their best performance [28]. Indirectly, the effects of presenteeism can be evaluated in this study using specific questionnaire items and register data. However, due to the absence of readily available indicators to apply here, a more comprehensive examination of the issue may be reserved for future studies.

We are aware that some parts of the accreditation are demanding and require going over the allotted work-hour resources. The hospital may have to spend additional time in ensuring the smooth operation of new formats. There might be a need for extra personnel for accreditation-related work. We have the approval from the hospital management and the commitment of the staff to this endeavor.

Numerous confounding factors exist. Health care is greatly influenced by cultural norms and is strictly regulated by legislation. The strength of qualitative studies is to raise points of view that are formulated through study questions. The qualitative approach in research is exploratory and helps develop hypotheses for future studies. To the best of the authors’ knowledge, the combination of the qualitative and quantitative approaches (ie, mixed methods) has not been used in research on hospital accreditation.

### Limitations

This protocol has certain limitations. The study will be a monocentric study; thus, comparative data from other hospitals are not collected. The lack of a comparative design is a limiting factor in the study protocol. However, comparing 2 hospitals, one with quality assurance implementation and the other without, presents challenges. These 2 hospitals have different personnel, patient profiles, and managerial routines as well as different practices and philosophies in quality assurance work. Probably all the confounding factors cannot be considered in this planned comparison. To overcome the lack of a comparative design, we are planning to apply a mixed methods approach that includes quantitative (questionnaires and register data) and qualitative (semistructured interviews) methods. The study also uses a convergent design [17], where both qualitative and quantitative data are collected as well as analyzed and results are reported at the same time.

The application of a locally developed questionnaire can be considered a limitation of the study. We argue, however, that it is well reasoned to apply this occupational well-being questionnaire in the planned study. The results would facilitate the development of Orton Orthopedic Hospital presumably more accurately than using a generic questionnaire. The whole concept of occupational well-being is novel and strongly influenced by cultural factors, supporting the use of local applications [25]. It is also worth noticing that when we compared the Parker and Hyett [29] questionnaire with ours, there were multiple similarities. Personnel turnover is also a limiting factor to be considered in the analyses.

## Appendix

Multimedia Appendix 1 Occupational well-being questionnaire.

## Abbreviations

JCI: Joint Commission International

## Data Availability

The data sets generated and analyzed in this study are not publicly available due to data access but are available from the corresponding author upon reasonable request.
