# Supplementary material for: The Effects of Quality Assurance System Implementation on Work Well-Being and Patient Safety: Protocol for a Mixed Methods Study
Source: JMIR Res Protoc. 2023 Nov 23;12:e45200. doi: 10.2196/45200 (PMC10704332; doi:10.2196/45200)
Supplement: Multimedia Appendix 1 [file resprot_v12i1e45200_app1.pdf]

Questionnaire: wellbeing at work

1. Gender
   a. male
   b. female
   c. other

–

2. Age (years)
   a. Under 25
   b. 25-29
   c. 30-44
   d. 45-54
   e. 55 or over

–

3. Duration of employment (years)
   a. under 1 year
   b. 1-5
   c. 6-10
   d. Over 10

–

4. Are you a supervisor?
   a. yes
   b. no

–

5. Employment
   a. fulltime
   b. part-time

–

6. For how many years have you attended a school? Please insert the answer in years, starting from the 1st grade of elementary school. : ________________________

–

7. Stress and load at current position (1 = extremely light ja 10 = extremely heavy)

	1 ⓘ	2	3	4	5	6	7	8	9	10 ⓘ
How is your work physically?	○	○	○	○	○	○	○	○	○	○
How is your work mentally?	○	○	○	○	○	○	○	○	○	○

8. Please estimate the amount that your illnesses and conditions have affected performing chores at home <u>during last month</u>. (1 = not at all ja 10 = I haven't been able to perform chores)

	1 ⓘ	2	3	4	5	6	7	9	8	10 ⓘ
	○	○	○	○	○	○	○	○	○	○

9. Health and mood

	Very good	Good	Average	Bad	Very bad
How has your health been during last month?	○	○	○	○	○
How has your mood been during last month?	○	○	○	○	○

10. How would you regard your health?
    a. Excellent
    b. Very good
    c. Good
    d. Satisfactory
    e. Bad

–

11. How well do the following statements define your situation? Insert your answers after each question. (1 = very much true and 5 = not at all true
    a. I feel that I get sick easier than most people ______
    b. I am atleast as healthy as the people I know _______
    c. I believe that my health condition will deteriorate ________
    d. My health is excellent ________

–

12. How rested and energetic have you been during last month?
    a. Very rested and full of energy
    b. Quite rested and energetic
    c. Not sure if energetic or exhausted
    d. Quite exhausted and tired
    e. Very exhausted and tired: I have been unable to conduct my tasks

–

13. How many workdays you had or had had during last month? Do not reduce days of absence. ______________________

–

14. How many days have you been absent from you work due to illnesses during last month? If you did not miss any days, mark "0". __________________

–

15. During the last 12 months, have you been at work while sick?
    a. Yes (insert number of days _________)
    b. No

–

16. Performance at work (1 = not at all and 10 = not able to perform)

	1	2	3	4	5	6	9	7	8	10
How much have your illnesses affected your performance at work during the last month?	○	○	○	○	○	○	○	○	○	○
Estimate how much your illnesses will affect your performance	○	○	○	○	○	○	○	○	○	○

at work in the next month.

17. Physical activity
    a. I have no difficulty in walking
    b. I have some difficulties in walking
    c. I am bedridden

    _

18. Personal hygiene
    a. I have no difficulty in taking care of myself
    b. I have some difficulties in showering or dressing myself
    c. I am unable to shower or dress myself

    _

19. Ordinary activities (work, studies, free time)
    a. I have no difficulties in completing ordinary activities
    b. I have some difficulties in completing ordinary activities
    c. I am unable to complete ordinary activities

    _

20. Pain and discomfort
    a. I have no pain or discomfort
    b. I have some pain or discomfort
    c. I have considerable pain or discomfort

    _

21. Anxiety and depression
    a. I am not anxious or depressed
    b. I am quite anxious or depressed
    c. I am very anxious or depressed

    _

	Completely agree	Somewhat agree	Do not agree nor disagree	Somewhat disagree	Completely disagree
22. Workload in my unit has generally been reasonable	1	2	3	4	5
23. I have a good work – life balance	1	2	3	4	5
24. Equipment in my unit is adequate	1	2	3	4	5
25. Work is flexible depending on the situation	1	2	3	4	5

	in my life					
26.	The physical load of my work is adequate	1	2	3	4	5
27.	The mental load of my work is adequate	1	2	3	4	5
28.	I trust that I can get help in problems regarding my ability to work	1	2	3	4	5
29.	Workplace safety is adequate	1	2	3	4	5
30.	There is no bullying at work	1	2	3	4	5
31.	There is no sexual harassment at work	1	2	3	4	5
32.	My superior has the courage to react to problems if needed	1	2	3	4	5
33.	I can constantly develop myself and my skills	1	2	3	4	5
34.	The values of our company realize well in daily operations	1	2	3	4	5
35.	We have a strong team spirit at work	1	2	3	4	5
